# Adverse effects of polychemotherapy for leprosy in 13 years of follow-up at a university hospital^☆^^☆☆^

**DOI:** 10.1016/j.abd.2020.07.005

**Published:** 2021-01-29

**Authors:** Violeta Duarte Tortelly, Egon Luiz Daxbacher, Arles Martins Brotas, Sueli Carneiro

**Affiliations:** a Department of Dermatology, Hospital Universitário Pedro Ernesto, Rio de Janeiro, RJ, Brazil; b Department of Dermatology, Instituto Endocrinologia Diabetes, Rio de Janeiro, RJ, Brazil

**Keywords:** Clofazimine, Dapsone, Leprosy, Rifampicin

## Abstract

Leprosy is one of the neglected diseases in the world and Brazil is the second country with more cases. A retrospective study was conducted based on the medical records of 196 leprosy patients diagnosed during the course of 13 years at a university hospital. The aim was to describe the adverse effects of polychemotherapy, as well the most prevalent and most vulnerable populations. In the study, dapsone was the most implicated drug, especially in women, and the risk increased with age. The authors conclude that with this patient profile, greater vigilance should be taken regarding possible adverse effects, especially anemia.

## Introduction

The multidrug therapy (MDT) recommended by the World Health Organization (WHO) has dramatically changed the natural history of leprosy. It reduced the prevalence and development of antimicrobial resistance.^1^ A cohort of 371 leprosy patients was followed-up for 13 years to identify the sociodemographic profile and the most frequent adverse effects of MDT.

## Methods

In a retrospective study from 2002 to 2014, 371 leprosy patients were evaluated and the socio-demographic profile and adverse effects (AE) of the standard MDT were evaluated. Paucibacillary (PB) and multibacillary (MB) patients who underwent less than four and nine supervised doses, respectively, were excluded; 196 patients remained.

## Results

Of the 196 patients, 97 were males and 99 females; 41.32% were treated with MDT-PB, 53.05% with MDT-MB, and 5.61% with an alternative regimen (Table 1). A total of 61 adverse effects were reported in 56 patients, of whom 64.28% (36) were women. Three patients were under 15 years of age; 12 were over 70 years; and 41 (73.21%), between 16 and 69 years. Hemolytic anemia was the most frequent adverse reaction (73.20%), whether acute (30.35%) or chronic (42.85%); 83.3% of these cases were observed in women, mainly aged 45 years or over (63.3%). The chance of a patient with anemia being female was 2.14 times greater (OR = 2.14; 95% CI: 1.14–4.12). Sulfonic syndrome occurred in two patients, while methemoglobinemia was observed in one (Table 2). Gastrointestinal alterations such as anorexia, nausea, vomiting, diarrhea, or epigastric pain were observed in nearly one-fifth of the patients, mostly (72.2%) due to DDS. Two patients presented photosensitivity. The chance of an individual having an AE increased with each year of age (OR = 1.02; 95% CI 1.00–1.04). Nonetheless, no association was observed between type of AE and age.

**Table 1:** Characteristics of the 196 leprosy patients followed-up at the hospital.

Characteristics	Number	Percentage (%)
Gender		
Male	97	49.0
Female	99	51.0
Ethnicity		
White	84	42.9
Black	42	21.4
Asian	1	0.5
Mixed-race	64	32.7
Native Brazilian	0	0
Unknown	5	2.5
Age (years)		
11 – 20	24	18.05
21 – 30	33	24.81
31 – 40	34	25.57
41 – 50	13	9.77
51 – 60	11	8.27
61 – 70	5	3.76
> 70	13	9.77
Clinical presentation		
Undetermined	17	8.67
Tuberculoid	61	31.12
Borderline	55	28.06
Lepromatous	60	30.61
Not classified	3	1.51
Educational status		
Illiterate	9	4.6
Elementary school	90	45.9
Incomplete high school	30	15.3
Complete high school	28	14.3
Incomplete university or college education	5	2.6
Complete university or college education	12	6.1
Unknown	19	9.7
Not applicable	3	1.5
Bacilloscopy		
Positive	80	40.8
Negative	94	48.0
Not performed	19	9.7
Unknown	3	1.5
Initial treatment		
MDT-PB	81	41.32
MDT-MB	104	53.06
Alternative	11	0.51
TOTAL	196	100%

MDT-PB, paucibacillary multidrug therapy; MDT-MB, multibacillary multidrug therapy.

**Table 2:** Characteristics of the 56 patients who had adverse effects.

Characteristics	Number	(%)
Gender		
Male	20	35.71
Female	36	64.29
Ethnicity		
White	27	48.21
Black	8	14.29
Asian	1	1.79
Mixed-race	18	32.14
Native Brazilian	0	0
Unknown	2	3.57
Age		
00 -15	3	5.36
16 – 45	22	39.28
46 – 69	19	33.93
≥ 70	12	21.43
Clinical presentation		
Indeterminate	6	10.71
Tuberculoid	15	26.79
Borderline	21	37.50
Lepromatous	13	23.21
Not classified	1	1.79
Education		
Illiterate	1	1.79
Incomplete fourth grade	12	21.43
Complete fourth grade	3	5.36
Incomplete elementary school	5	8.93
Complete elementary school	1	1.79
Incomplete high school	10	17.85
Complete high school	10	17.85
Incomplete university or college education	1	1.79
Complete university or college education	6	10.71
Unknown	5	8.93
Not applicable	2	3.57
Bacilloscopy		
Positive	20	35.71
Negative	30	53.58
Not performed	6	10.71
Adverse effect dose		
First	19	33.93
Second	9	16.07
Third	9	16.07
Fourth	6	10.71
Fifth or greater	13	23.21
Initial treatment		
MDT-PB	21	37.50
MDT-MB	35	62.50
TOTAL	56	100

MDT-PB, paucibacillary multidrug therapy; MDT-MB, multibacillary multidrug therapy.

Most AEs occurred in the first few supervised doses, 33.92% in the first and 76.78% until the fourth (Table 2). DDS was the most implicated drug; it accounted for 96.7% of AE (59/61), and was replaced in almost half of the cases (49.15%). RFP alone represented 3.27% of the AEs and was suspended in only one patient, due to refractory headache. Mild thrombocytopenia occurred in three patients using a combination of RFP and DDS. Some patients presented more than one AE (Table 3).

**Table 3:** Adverse effects, respective drugs, and suspension.

Adverse effect due to dapsone	n	Dapsone suspension
Chronic hemolytic anemia	24	11
Acute hemolytic anemia	17	12
Photosensitivity/photodermatitis	2	2
Methemoglobinemia	1	1
Gastrointestinal manifestation	8	0
Dapsone hypersensitivity syndrome	3	3
**Adverse effect due to rifampicin**	**n**	**Rifampicin suspension**
Gastrointestinal manifestation	1	0
Headache	1	1
**Adverse effect due to clofazimine and dapsone**	**n**	**Dapsone or clofazimine suspension**
Gastrointestinal manifestation	1	0
**Adverse effect due to rifampicin and dapsone**	**n**	**Dapsone or rifampicin suspension**
Thrombocytopenia	3	0
Total adverse effects	61	30

## Discussion

In the cohort of 196 individuals studied, most MB treatments were in male patients. Nobre et al. reported twice as much MB cases in men (OR = 2.36; 95% CI 2.33–2.38).^2^ Half of the patients were between 20 and 40 years of age. Literature data show that AE can occur in up to half of the patients (45%) in MDT; in the present study, it occurred in less than 30%.^3^, ^4^, ^5^ Although more than a quarter (n = 56) of the patients experienced an AE, the drug was replaced in 30 of the 185 cases that started the standard regimen. More than two-thirds of AEs occurred in the first four months of treatment and DDS was the medication most involved, according to reports in the literature.^5^ Hematological AE occurred in almost three quarters of the patients and have been related to the individual capacity for sulfamine (DDS) acetylation and hydroxylation. Individuals with hemoglobinopathies, G6PD deficiency, and those receiving oxidative medications may be at increased risk for hemolytic anemia.^6^ Dapsone-related photoallergy has led to the discontinuation of the drug in two cases. The occurrence of AE in women was twice as high, corroborating Dupnik's findings of the association of female gender with an increased risk of drug reaction.^7^ G6PD genes are on the X chromosome and some mutations lead women with partial disabilities to develop symptoms.^8^ In the present study, it was observed that women over 45 years of age had a higher risk of AE, contrary to the findings by Dupnik, who observed it in younger women.^7^ The chance of an individual having AE increases with each year of age for both sexes (OR = 1.02; 95% CI 1.00–1.04). Slower metabolization in older patients would justify this finding.^9^ Goulart et al. also found an increased risk of hematological effects in older patients.^3^ Sulfonic syndrome, although severe, is uncommon; it was observed in 1.53%, in agreement with the literature.^3^, ^5^, ^10^ Gastrointestinal complaints improved with recommendations for concomitant food intake and/or use of antiemetics, not requiring drug suspension.

## Conclusions

DDS was responsible for the largest number of AE cases, mainly in the first trimester. Anemia was the most frequent, mainly in women. The risk of AE increases with each year of age. These findings reinforce the need for greater vigilance at the beginning of treatment, in women, and in older patients.

## Financial support

None declared.

## Authors’ contributions

Violeta Duarte Tortelly: Approval of the final version of the manuscript; design and planning of the study; drafting and editing of the manuscript; collection, analysis, and interpretation of data; effective participation in research orientation; intellectual participation in propaedeutic and/or therapeutic conduct of studied cases; critical review of the literature; critical review of the manuscript.

Egon Luiz Daxbacher: Approval of the final version of the manuscript; design and planning of the study; effective participation in research orientation; critical review of the literature.

Arles Martins Brotas: Approval of the final version of the manuscript; critical review of the literature; critical review of the manuscript.

Sueli Carneiro: Approval of the final version of the manuscript; design and planning of the study; drafting and editing of the manuscript; critical review of the manuscript.

## Conflicts of interest

None declared.
